# A Physics-Guided Machine Learning Model for Predicting Viscoelasticity of Solids at Large Deformation

**DOI:** 10.3390/polym16223222

**Published:** 2024-11-20

**Authors:** Bao Qin, Zheng Zhong

**Affiliations:** 1 Research Institute of Interdisciplinary Science, School of Materials Science and Engineering, Dongguan University of Technology, Dongguan 523808, China; qinbao@dgut.edu.cn; 2 School of Science, Harbin Institute of Technology, Shenzhen 518055, China

**Keywords:** machine learning, viscoelasticity, large deformation, recurrent neural networks, polymers

## Abstract

Physics-guided machine learning (PGML) methods are emerging as valuable tools for modelling the constitutive relations of solids due to their ability to integrate both data and physical knowledge. While various PGML approaches have successfully modeled time-independent elasticity and plasticity, viscoelasticity remains less addressed due to its dependence on both time and loading paths. Moreover, many existing methods require large datasets from experiments or physics-based simulations to effectively predict constitutive relations, and they may struggle to model viscoelasticity accurately when experimental data are scarce. This paper aims to develop a physics-guided recurrent neural network (RNN) model to predict the viscoelastic behavior of solids at large deformations with limited experimental data. The proposed model, based on a combination of gated recurrent units (GRU) and feedforward neural networks (FNN), utilizes both time and stretch (or strain) sequences as inputs, allowing it to predict stress dependent on time and loading paths. Additionally, the paper introduces a physics-guided initialization approach for GRU–FNN parameters, using numerical stress–stretch data from the generalized Maxwell model for viscoelastic VHB polymers. This initialization is performed prior to training with experimental data, helping to overcome challenges associated with data scarcity.

## 1. Introduction

Machine learning has made significant strides in various fields of science and engineering, driven by the proliferation of digital data, increasing computing power, and advanced algorithms. It has achieved notable success in applications such as speech recognition [1], image classification [2], and cognitive science [3]. Recently, machine learning has emerged as a promising tool for predicting deformation patterns [4,5] by modeling the mechanical constitutive behavior of solids, particularly the relationship between mechanical stress and strain. However, modeling both time and history-dependent nonlinear constitutive relations such as viscoelasticity presents challenges for two reasons. First, many state-of-the-art machine learning techniques (e.g., feedforward/convolutional/recurrent neural networks, FNN/CNN/RNN) [6,7,8] require large datasets and often lack robustness, failing to guarantee convergence when only limited experimental stress–strain data are available from constitutive experiments, such as uniaxial tensile or bending tests. Second, modelling time and history-dependent nonlinear stress–strain relations can be difficult for many machine learning techniques, including the popular FNN, due to their limited capacity to handle sequential information. Therefore, advancing machine learning approaches to effectively model viscoelasticity in solids at large deformation is crucial for furthering the study of solid mechanics problems.

Physics-guided machine learning (PGML) methods, which integrate established physical principles with data-driven approaches, have proven beneficial in solid mechanics by simultaneously incorporating data information and physical knowledge [9,10,11]. These studies can be broadly classified into three categories: (i) discovering constitutive equations from data [12,13,14,15,16,17]; (ii) solving governing equations [18,19,20,21,22]; (iii) predicting data-driven constitutive relations [15,23,24,25,26,27,28]. Here, we focus on elaborating the third category. To predict data-driven constitutive relations, significant efforts have been made using deep neural networks, including FNN, temporal convolutional network (TCN), and RNN, among others. For example, Linka et al. [15] proposed a data-driven constitutive neural network model for predicting the mechanical constitutive behavior of hyperelastic materials. This model integrates physical laws and the symmetry of mechanical properties into the network structure, enabling the simultaneous use of information from three sources: stress–strain data, theoretical knowledge from materials science, and additional data such as microstructural or processing information; Danoun et al. [23] developed a thermodynamically consistent RNN model to simulate the mechanical response of elastoplastic materials under multi-axial and non-proportional loading conditions. Their work demonstrates that incorporating thermodynamic consistency can significantly enhance the predictive capabilities of such surrogate models; Wang et al. [24] developed a TCN model for materials with an ultra-long-history-dependent stress–strain relation. Their TCN model outperforms RNN models, including Long Short-Term Memory (LSTM) and Gated Recurrent Unit (GRU) models, in terms of both performance and speed for various sequential tasks. Abdolazizi et al. [28] proposed viscoelastic Constitutive Artificial Neural Networks (vCANNs), a novel physics-informed machine learning framework for anisotropic nonlinear viscoelasticity at finite strains. This approach is based on generalized Maxwell models, enhanced with neural networks to capture nonlinear strain- and strain rate-dependent properties. However, most of these models require a moderate to large amount of labeled data from experiments or physics-based simulations to accurately learn time-independent constitutive relations, such as those for path-independent elastic materials and path-dependent plastic materials [26,27]. They often face challenge in modelling the viscoelasticity of solids, which depends on both time and loading paths, especially when experimental data are scarce. Additionally, incorporating physical knowledge of viscoelasticity, such as viscous dissipation energy, directly into the training process is challenging. This difficulty arises because instantaneous viscous deformation and its conjugated driving force, two key components for calculating viscous dissipation energy, are difficult to obtain through experiments.

Therefore, this paper aims to develop a physics-guided machine learning model for predicting the viscoelasticity of solids at large deformation with scarce experimental data. First, we propose a surrogate model of viscoelasticity based on GRU and FNN (GRU–FNN). This model inputs both time and stretch (or strain) sequences, enabling it to predict stress based on time and loading path. Second, we implement a physics-guided initialization for the GRU–FNN parameters by training on numerical stress–stretch data derived from the generalized Maxwell model for viscoelastic solids under large deformations. This approach allows the model to predict the viscoelastic behavior of solids even with scarce experimental stress–stretch data. Unlike existing data-driven models that use residuals of the governing equations or variational forms as penalizing terms to restrict the solution space, our strategy effectively avoids the need for direct measurements of viscous deformation.

The remainder of this paper is organized as follows. In Section 2, a continuum theoretical framework for the viscoelasticity of solids at large deformation is given and constitutive equations including the state and evolving equations are derived. In Section 3, a GRU–FNN model for viscoelasticity is established. In Section 4, uniaxial viscoelasticity of commercially available dielectric polymers VHB4905 is modelled. Finally, conclusions are given in Section 5.

## 2. Thermodynamic Formulation of Constitutive Laws for Viscoelastic Solids at Large Deformation

Consider a solid $B_{0}$ bounded by the surface $\partial B_{0}$, defined in a fixed reference configuration, which deforms into $B_{t}$ with the surface $\partial B_{t}$. The deformation gradient is then given by

(1) $$F=\nabla_{R}x$$

**Figure 1:**
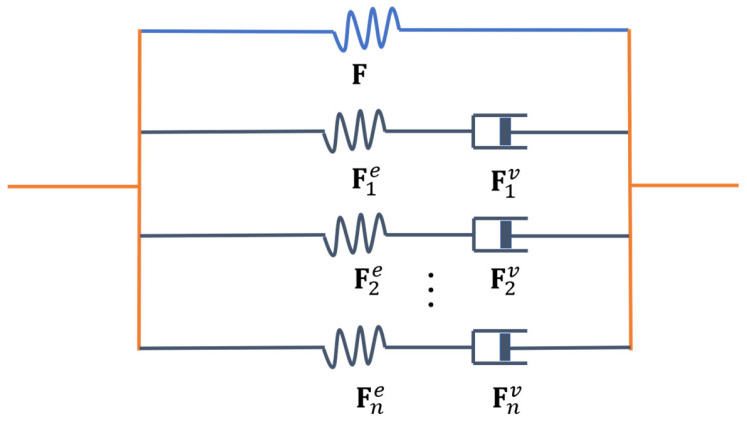
The generalized Maxwell model for solids under large deformation. It is assumed to be equivalent to an equilibrium spring with a deformation gradient ***F***, and *n* parallel Maxwell element, each characterized by an elastic deformation gradient $F_{i}^{e}$ and a viscous deformation gradient $F_{i}^{v}$ (1 ≤ *i* ≤ *n*).

Here, $x=\chi \left(X,t\right)$ is a function that maps an arbitrary material point X inside $B_{0}$ into a spatial point x inside $B_{t}$, and $\nabla_{R}$ represents the gradient operator with respect to the coordinates X. To describe the viscoelasticity of solids under large deformation, a generalized Maxwell model [29,30,31,32,33] is employed, as shown in Figure 1. In this model, the solid is represented by an equilibrium spring, characterized by the deformation gradient F, along with n parallel Maxwell elements (each consisting of a spring and a dashpot in series), characterized by the elastic deformation gradient $F_{i}^{e}$ and the viscous deformation gradient $F_{i}^{v}$, leading to the following relation:

(2) $$F=F_{i}^{e}\cdot F_{i}^{v},\;i=1,\ldots ,n$$

Accordingly, the right Cauchy–Green deformation tensors, $C=F^{T}\cdot F$ and $C_{i}^{e}={\left(F_{i}^{e}\right)}^{T}\cdot F_{i}^{e}$, where the superscript T denotes the transpose, are used to measure the deformation of the equilibrium spring and the elastic deformation of the Maxwell elements, respectively.

Neglecting inertial effects, the balance laws of force and moment in the current configuration are given by

(3) $$\sigma \cdot \nabla +b=0,\;\sigma =\sigma^{T}$$

where σ is the Cauchy stress tensor and b is the body force per unit volume in $B_{t}$, which can be expressed in the reference configuration as

(4) $$P\cdot \nabla_{R}+b_{R}=0,\;P\cdot F^{T}=F\cdot P^{T}$$

where P denotes the first P–K stress tensor and $b_{R}$ denotes the body force per unit volume in $B_{0}$. Let j denote the determinant of F, and we have the relations $P=j\sigma \cdot {\left(F^{T}\right)}^{-1}$ and $b_{R}=jb$.

Let ε and q represent the internal energy density and the heat source per unit volume in $B_{0}$, respectively, and $j^{q}$ denote the heat flux per unit area on $\partial B_{t}$. The energy balance law in the current configuration is then expressed as

(5) $$\dot{\varepsilon }=\sigma :\nabla \dot{\chi }-\nabla \cdot j^{q}+q$$

whose corresponding form in the reference configuration is

(6) $${\dot{\varepsilon }}_{R}=P:\dot{F}-\nabla_{R}\cdot j_{R}^{q}+q_{R}$$

where $\frac{d}{dt}$ or equivalently a superposed dot represents the material time derivative. $\varepsilon_{R}$ and $q_{R}$ denote the internal energy density and the heat source per unit volume in $B_{0}$, respectively, while $j_{R}^{q}$ represents the heat flux per unit area on $\partial B_{0}$. The relationships $\varepsilon_{R}=j\varepsilon$, $q_{R}=jq$ and $j_{R}^{q}=jj^{q}\cdot {\left(F^{T}\right)}^{-1}$ hold for the above two equations. The first term on the right-hand side represents the mechanical work, while the second and third terms account for the energy from heat flow across the surface and the heat source inside the body, respectively.

Let η denote the entropy per unit volume in $B_{t}$ and ϑ the absolute temperature. The entropy inequality in the current configuration is expressed locally as

(7) $$\dot{\eta }\geq -\nabla \cdot \frac{j^{q}}{\vartheta }+\frac{q}{\vartheta }$$

which can also be written in the reference configuration as

(8) $${\dot{\eta }}_{R}\geq -\nabla_{R}\cdot \frac{j_{R}^{q}}{\vartheta }+\frac{q_{R}}{\vartheta }$$

where $\eta_{R}$ is the entropy per unit volume in $B_{0}$, related to η by $\eta_{R}=j\eta$.

Introducing the Helmholtz free energy density φ=ε−ϑη and considering the energy balance (5), the inequality (7) becomes

(9) $$\sigma :\nabla \dot{\chi }-\eta \dot{\vartheta }-\dot{\varphi }-\frac{1}{\vartheta }j^{q}\cdot \nabla \vartheta \geq 0$$

Similarly, by introducing the Helmholtz free energy density $\varphi_{R}=\varepsilon_{R}-\vartheta \eta_{R}$, where $\varphi_{R}=j\varphi$, and considering the energy balance (6), the inequality becomes

(10) $$P:\dot{F}-\eta_{R}\dot{\vartheta }-{\dot{\varphi }}_{R}-\frac{1}{\vartheta }j_{R}^{q}\cdot \nabla_{R}\vartheta \geq 0$$

This imposes the thermodynamic constraint on solids. For convenience, we will use the formulations provided in the reference configuration in the following sections.

Considering the thermo-viscoelastic effects in solids, the Helmholtz free energy density $\varphi_{R}$ can be assumed to be a function of variables $\left(C,C_{i}^{e},\vartheta \right)$, i.e.,

(11) $$\varphi_{R}=\varphi_{R}\left(C,C_{i}^{e},\vartheta \right)$$

whose material time derivative can be further written as

(12) $${\dot{\varphi }}_{R}=\frac{\partial \varphi_{R}}{\partial C}:\dot{C}+\sum_{i=1}^{n}\frac{\partial \varphi_{R}}{\partial C_{i}^{e}}:{\dot{C}}_{i}^{e}+\frac{\partial \varphi_{R}}{\partial \vartheta }\dot{\vartheta }$$

The first and second terms on the right-hand side of Equation (12) can be rewritten as

(13) $$\begin{array}{l} \frac{\partial \varphi_{R}}{\partial C}:\dot{C}=2F\cdot \frac{\partial \varphi_{R}}{\partial C}:\dot{F}\\ \frac{\partial \varphi_{R}}{\partial C_{i}^{e}}:{\dot{C}}_{i}^{e}=\frac{\partial \varphi_{R}}{\partial C_{i}^{e}}:\frac{\partial C_{i}^{e}}{\partial F}:\dot{F}+\frac{\partial \varphi_{R}}{\partial C_{i}^{e}}:\frac{\partial C_{i}^{e}}{\partial F_{i}^{v}}:{\dot{F}}_{i}^{v}=2F_{i}^{e}\cdot \frac{\partial \varphi_{R}}{\partial C_{i}^{e}}\cdot {\left(F_{i}^{vT}\right)}^{-1}:\dot{F}-2C_{i}^{e}\cdot \frac{\partial \varphi_{R}}{\partial C_{i}^{e}}:D_{i}^{v} \end{array}$$

with

(14) $$D_{i}^{v}=\frac{1}{2}\left(L_{i}^{v}+L_{i}^{vT}\right),\;L_{i}^{v}={\dot{F}}_{i}^{v}\cdot {\left(F_{i}^{v}\right)}^{-1}$$

where the superscript ‘−1’ denotes the inverse of a tensor. Then, substitution of Equations (12) and (13) into Equation (10) yields

(15) $$\left(P-2F\cdot \frac{\partial \varphi_{R}}{\partial C}-2F_{i}^{e}\cdot \frac{\partial \varphi_{R}}{\partial C_{i}^{e}}\cdot {\left(F_{i}^{vT}\right)}^{-1}\right):\dot{F}-\left(\eta_{R}+\frac{\partial \varphi_{R}}{\partial \vartheta }\right)\dot{\vartheta }-\frac{1}{\vartheta }j_{R}^{q}\cdot \nabla_{R}\vartheta +\sum_{i=1}^{n}2C_{i}^{e}\cdot \frac{\partial \varphi_{R}}{\partial C_{i}^{e}}:D_{i}^{v}\geq 0$$

In the case where P and $\eta_{R}$ are independent of $\dot{F}$ and $\dot{\vartheta }$, the first two terms of the above inequality must vanish, leading to the following constitutive relations:

(16) $$P=2F\cdot \frac{\partial \varphi_{R}}{\partial C}+2F_{i}^{e}\cdot \frac{\partial \varphi_{R}}{\partial C_{i}^{e}}\cdot {\left(F_{i}^{vT}\right)}^{-1}$$

(17) $$\eta_{R}=-\frac{\partial \varphi_{R}}{\partial \vartheta }$$

Thus, the inequality (15) reduces to

(18) $$-\frac{1}{\vartheta }j_{R}^{q}\cdot \nabla_{R}\vartheta +\sum_{i=1}^{n}M_{i}^{neq}:D_{i}^{v}\geq 0$$

where $M_{i}^{neq}$ is the non-equilibrium Mandel stress tensor [34], defined as

(19) $$M_{i}^{neq}=2C_{i}^{e}\cdot \frac{\partial \varphi_{R}}{\partial C_{i}^{e}}$$

More specially, to satisfy the thermodynamic constraint imposed by inequality (18), the following constitutive equations are derived:

(20) $$j_{R}^{q}=-Y\cdot \nabla_{R}\vartheta$$

(21) $$D_{i}^{v}=Q_{i}:M_{i}^{neq}$$

where Y is a second order tensor and $Q_{i}$ is a fourth order tensor, both of which are positive-definite. Here, Equation (20) represents the Fourier heat conduction law, and Equation (21) describes the rheological viscous flow rule [30,34].

The Helmholtz free energy density is assumed to be additively decomposed as follows:

(22) $$\varphi_{R}=\varphi_{R}^{eq}+\varphi_{R}^{neq}$$

where $\varphi_{R}^{eq}$ and $\varphi_{R}^{neq}$ represent the equilibrium and non-equilibrium Helmholtz free energy densities, corresponding to the stretching of the single spring and the springs in Maxwell element, respectively. We adopt the Gent model [35] for both $\varphi_{R}^{eq}$ and $\varphi_{R}^{neq}$ to account for the strain-stiffening effect, where solids may stiffen sharply as the stretch approaches their extension limit [36], as follows:

(23) $$\varphi_{R}^{eq}=-\frac{G^{eq}L}{2}\ln\left(\frac{L-trC+3}{L}\right)$$

(24) $$\varphi_{R}^{neq}=-\sum_{i=1}^{n}\frac{G_{i}^{neq}L_{i}}{2}\ln\left(\frac{L_{i}-trC_{i}^{e}+3}{L_{i}}\right)$$

where the symbol ‘tr’ denotes the trace of a tensor, $G^{eq}$ and $G_{i}^{neq}$ represent the equilibrium modulus of the single spring and the nonequilibrium modulus of the *i*th Maxwell element, respectively, L and $L_{i}$ denote the extension limits of the single spring and the *i*th Maxwell element spring, respectively. Substituting Equations (22) into (16) and (19), we have

(25) $$P=\frac{G^{eq}L}{L-trC+3}F+\sum_{i=1}^{n}\frac{G_{i}^{neq}L_{i}}{L_{i}-trC_{i}^{e}+3}F_{i}^{e}\cdot {\left(F_{i}^{vT}\right)}^{-1}$$

(26) $$M_{i}^{neq}=\frac{G_{i}^{neq}L_{i}}{L_{i}-trC_{i}^{e}+3}C_{i}^{e}$$

Next, consider a case that the elastic deformations of springs are incompressible, which generally applies to polymers [32]. Lagrange multipliers Π and $\Pi_{i}$ are introduced to enforce these constraint conditions, modifying the free energy density as

(27) $${\tilde{\varphi }}_{R}=\varphi_{R}-\Pi \left(j-1\right)-\sum_{i=1}^{n}\Pi_{i}\left(j_{i}^{e}-1\right)$$

where $j_{i}^{e}$ is the determinant of the elastic deformation gradient $F_{i}^{e}$. Replacing $\varphi_{R}$ with ${\tilde{\varphi }}_{R}$ in Equations (16) and (19), we can rewrite P and $M_{i}^{neq}$ as

(28) $$P=\frac{G^{eq}L}{L-trC+3}F+\sum_{i=1}^{n}\frac{G_{i}^{neq}L_{i}}{L_{i}-trC_{i}^{e}+3}F_{i}^{e}\cdot {\left(F_{i}^{vT}\right)}^{-1}-j\Pi {\left(F^{T}\right)}^{-1}-\sum_{i=1}^{n}j_{i}^{e}\Pi_{i}{\left(F^{T}\right)}^{-1}$$

(29) $$M_{i}^{neq}=\frac{G_{i}^{neq}L_{i}}{L_{i}-trC_{i}^{e}+3}C_{i}^{e}-j_{i}^{e}\Pi_{i}I$$

where I is the second-order unit tensor. Furthermore, the Cauchy stress tensor σ can be obtained using the relations $\sigma =\frac{1}{j}P\cdot F^{T}$, and $j=j_{i}^{e}=1$ in Equation (28), as follows:

(30) $$\sigma =\frac{G^{eq}L}{L-trC+3}F\cdot F^{T}+\sum_{i=1}^{n}\frac{G_{i}^{neq}L_{i}}{L_{i}-trC_{i}^{e}+3}F_{i}^{e}\cdot F_{i}^{eT}-\left(\Pi +\sum_{i=1}^{n}\Pi_{i}\right)I$$

By employing $j=j_{i}^{e}=1$ and the multiplicative decomposition of F from Equation (2), the condition of viscous incompressibility, $trD_{i}^{v}=0$ or $j_{i}^{v}=1$, where $j_{i}^{v}$ is the determinant of $F_{i}^{v}$, can be deduced. From this, the fourth-order tensor $Q_{i}$ in Equation (21) can be expressed as [32]

(31) $$Q_{i}=\frac{1}{2v_{i}}\left(I_{4}-\frac{1}{3}I⊗I\right)$$

where $I_{4}$ is the fourth-order unit tensor and $v_{i}$ is the viscosity of the ith subnetwork. The relaxation time for the ith Maxwell element is then defined as $\tau_{i}=\frac{v_{i}}{G_{i}^{neq}}$ [31].

The corresponding deformation gradient in tensile tests, without shear deformation, can be expressed as

(32) $$\begin{array}{l} F=\left[\begin{array}{ccc} \lambda_{1} & 0 & 0\\ 0 & \lambda_{2} & 0\\ 0 & 0 & \lambda_{3} \end{array}\right],\;F_{i}^{e}=\left[\begin{array}{ccc} \lambda_{i1}^{e} & 0 & 0\\ 0 & \lambda_{i2}^{e} & 0\\ 0 & 0 & \lambda_{i3}^{e} \end{array}\right],\;F_{i}^{v}=\left[\begin{array}{ccc} \lambda_{i1}^{v} & 0 & 0\\ 0 & \lambda_{i2}^{v} & 0\\ 0 & 0 & \lambda_{i3}^{v} \end{array}\right]\\ D_{i}^{v}=\left[\begin{array}{ccc} {\dot{\lambda }}_{i1}^{v}/\lambda_{i1}^{v} & 0 & 0\\ 0 & {\dot{\lambda }}_{i2}^{v}/\lambda_{i2}^{v} & 0\\ 0 & 0 & {\dot{\lambda }}_{i3}^{v}/\lambda_{i3}^{v} \end{array}\right] \end{array}$$

Here $\lambda_{1}$, $\lambda_{2}$, $\lambda_{3}$ are the principal stretches of the deformation gradient F; $\lambda_{i1}^{e}$, $\lambda_{i2}^{e}$, $\lambda_{i3}^{e}$ are the principle stretches of the elastic deformation gradient $F_{i}^{e}$; and $\lambda_{i1}^{v}$, $\lambda_{i2}^{v}$, $\lambda_{i3}^{v}$ are the principal stretches of the viscous deformation gradient $F_{i}^{v}$. Additionally, considering the equal lateral stretches during the uniaxial tensile test and the incompressible deformation condition $j=j_{i}^{e}=j_{i}^{v}=1$, we have

$$\lambda_{1}=\lambda ,\;\lambda_{2}=\lambda_{3}={\left(\lambda \right)}^{-\frac{1}{2}}$$

$$\;\lambda_{1}=\lambda_{i1}^{e}\lambda_{i1}^{v}=\lambda ,\;\lambda_{i2}^{e}\lambda_{i2}^{v}=\lambda_{i3}^{e}\lambda_{i3}^{v}={\left(\lambda \right)}^{-\frac{1}{2}}$$

(33) $$\lambda_{i1}^{e}=\lambda_{i}^{e},\;\lambda_{i2}^{e}=\lambda_{i3}^{e}={\left(\lambda_{i}^{e}\right)}^{-\frac{1}{2}},\;\lambda_{i1}^{v}=\lambda_{i}^{v},\;\lambda_{i2}^{v}=\lambda_{i3}^{v}={\left(\lambda_{i}^{v}\right)}^{-\frac{1}{2}}$$

with λ, $\lambda_{i}^{e}$, $\lambda_{i}^{v}$ being the principal stretches of the deformation gradients F, $F_{i}^{e}$, $F_{i}^{v}$ along the tensile direction, respectively.

Let $P_{1}$, $P_{2}$ $P_{3}$ denote the components of P along the three principal directions, respectively. Substituting Equations (32) and (33) into Equation (28), we obtain

(34) $$\begin{array}{l} P_{1}=\frac{G^{eq}L\lambda }{L-2\lambda^{-1}-\lambda^{2}+3}+\sum_{i=1}^{n}\frac{G_{i}^{neq}L_{i}\lambda {\left(\lambda_{i}^{v}\right)}^{-2}}{L_{i}-2\lambda^{-1}\lambda_{i}^{v}-\lambda^{2}{\left(\lambda_{i}^{v}\right)}^{-2}+3}-\frac{\Pi +\sum_{i=1}^{n}\Pi_{i}}{\lambda }\\ P_{2}=P_{3}=\frac{G^{eq}L\lambda^{-\frac{1}{2}}}{L-2\lambda^{-1}-\lambda^{2}+3}+\sum_{i=1}^{n}\frac{G_{i}^{neq}L_{i}\lambda^{-\frac{1}{2}}\lambda_{i}^{v}}{L_{i}-2\lambda^{-1}\lambda_{i}^{v}-\lambda^{2}{\left(\lambda_{i}^{v}\right)}^{-2}+3}-\left(\Pi +\sum_{i=1}^{n}\Pi_{i}\right)\lambda^{\frac{1}{2}} \end{array}$$

According to the boundary condition $P_{2}=P_{3}=0$ for uniaxial loading–unloading tests, we can further derive

(35) $$\Pi +\sum_{i=1}^{n}\Pi_{i}=\frac{G^{eq}L\lambda^{-1}}{L-2\lambda^{-1}-\lambda^{2}+3}+\sum_{i=1}^{n}\frac{G_{i}^{neq}L_{i}\lambda^{-1}\lambda_{i}^{v}}{L_{i}-2\lambda^{-1}\lambda_{i}^{v}-\lambda^{2}{\left(\lambda_{i}^{v}\right)}^{-2}+3}$$

and

(36) $$P_{1}=\frac{G^{eq}L\left(\lambda -\lambda^{-2}\right)}{L-2\lambda^{-1}-\lambda^{2}+3}+\sum_{i=1}^{n}\frac{G_{i}^{neq}L_{i}\left[\lambda {\left(\lambda_{i}^{v}\right)}^{-2}-\lambda^{-2}\lambda_{i}^{v}\right]}{L_{i}-2\lambda^{-1}\lambda_{i}^{v}-\lambda^{2}{\left(\lambda_{i}^{v}\right)}^{-2}+3}$$

Here, the effect of deformation incompressibility on the stress is considered, and the Lagrange multipliers are eliminated according to the boundary conditions.

Let $M_{i1}^{neq}$, $M_{i2}^{neq}$ $M_{i3}^{neq}$ denote the components of $M_{i}^{neq}$ along the three principal directions, respectively. Similarly, substituting Equations (32) and (33) into Equation (29), we obtain

(37) $$\begin{array}{l} M_{i1}^{neq}=\frac{G_{i}^{neq}L_{i}\lambda^{2}{\left(\lambda_{i}^{v}\right)}^{-2}}{L_{i}-2\lambda^{-1}\lambda_{i}^{v}-\lambda^{2}{\left(\lambda_{i}^{v}\right)}^{-2}+3}-\Pi_{i}\\ M_{i2}^{neq}=M_{i3}^{neq}=\frac{G_{i}^{neq}L_{i}\lambda^{-1}\lambda_{i}^{v}}{L_{i}-2\lambda^{-1}\lambda_{i}^{v}-\lambda^{2}{\left(\lambda_{i}^{v}\right)}^{-2}+3}-\Pi_{i} \end{array}$$

Then, substitution of Equations (37), (32) and (31) into Equation (21) yields

(38) $$\frac{{\dot{\lambda }}_{i}^{v}}{\lambda_{i}^{v}}=\frac{L_{i}}{3\tau_{i}\left[L_{i}-2\lambda^{-1}\lambda_{i}^{v}-\lambda^{2}{\left(\lambda_{i}^{v}\right)}^{-2}+3\right]}\left[\lambda^{2}{\left(\lambda_{i}^{v}\right)}^{-2}-\lambda^{-1}\lambda_{i}^{v}\right]$$

This describes the viscous flow in solids subjected to uniaxial tension. It is important to note that the elastic incompressibility of the springs does not affect the viscous flow, as only the deviatoric stress, excluding the hydrostatic pressure $\Pi_{i}$, derives the viscous flow.

**Figure 2:**
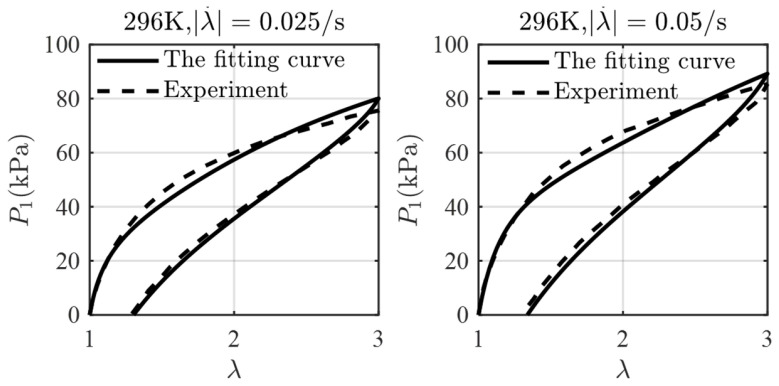
The fitting stress–stretch curves between theoretical model and experimental data.

**Figure 3:**
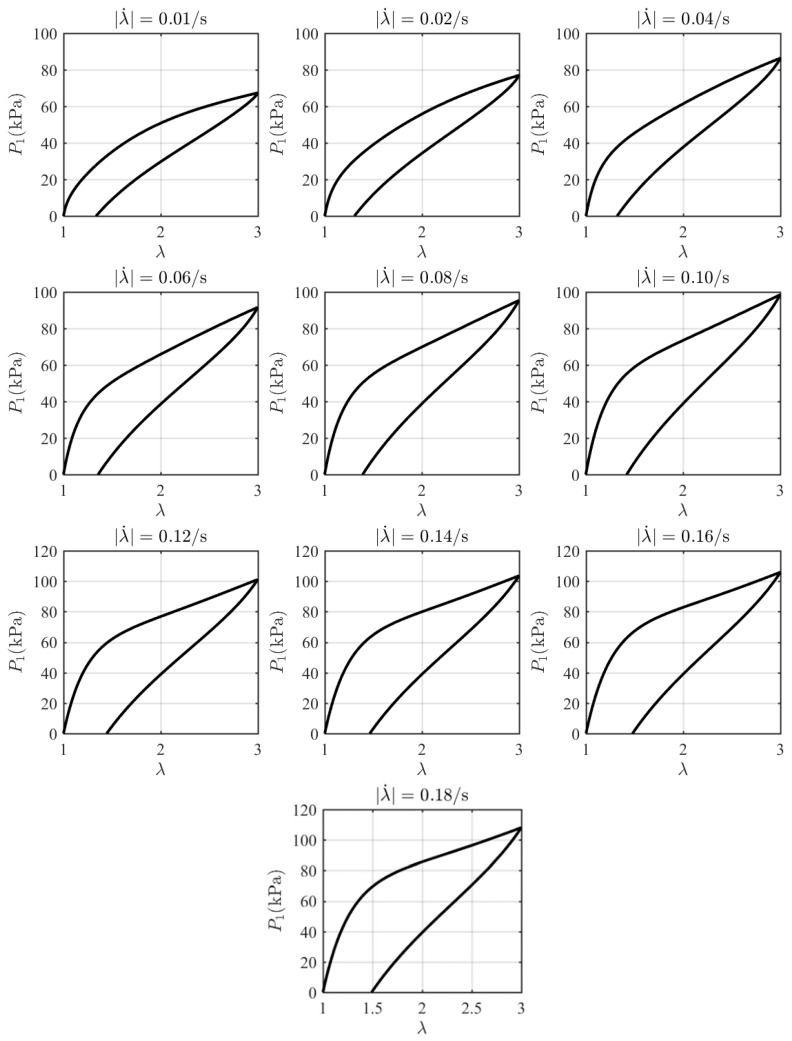
Theoretical stress–stretch curves at different stretching rates.

Under the initial conditions ${\left.P_{1}\right|}_{t=0}=0$, ${\left.\lambda \right|}_{t=0}={\left.\lambda_{i}^{v}\right|}_{t=0}=1$, the coupled Equations (36) and (38) can be solved simultaneously to predict the mechanical behaviors based on the given material parameters. A finite difference approach is used to obtain the numerical results for the evolving stress $P_{1}$ as a function of the stretch λ at ten different stretching rates. The model parameters are obtained by fitting Equations (36) and (38) with the experimental data at 296K as *n* = 3, $L=L_{i}=155\;\left(i=1,\;2,\;3\right)$, $G^{eq}=15.12\;\mathrm{kPa}$, $G_{1}^{neq}=16.83\;\mathrm{kPa}$, $G_{2}^{neq}=27.39\;\mathrm{kPa}$, $G_{3}^{neq}=41.04\;\mathrm{kPa}$, $\tau_{1}=413.32\;s$, $\tau_{2}=5.43s$, $\tau_{3}=1.65\;s$. Great consistency between the experimental data and the theoretical model is shown in Figure 2. With these parameters determined, the theoretical data at different stretching rates are calculated as shown in Figure 3. The stress can be observed to increase with stretch during the loading phase and decrease with stretch during the unloading phase. Each loading–unloading cycle exhibits characteristic viscoelastic behavior, with the peak stress magnitude rising as the stretching rate increases.

The initialization of parameters of the following machine learning model will be obtained by training these theoretical results, which is proven to be an effective strategy for predicting viscoelasticity of solids with scarce experimental data. This strategy avoids directly imposing physical constraints on the training procedure since the specific governing equations are usually uncertain and the physical quantities such as viscous deformation and its conjugated driving force are hard to obtain via experiments. In the next section, a machine learning model for predicting viscoelasticity will be introduced.

## 3. Machine Learning Method for Predicting Viscoelasticity

As mentioned, the FNN architecture is not well suited for handling history-dependent behaviors because there is no direct relationship between the network inputs at a current time step and the outputs from previous time steps. To address this issue, the RNN architecture is introduced as a reliable model for managing this history dependency. RNN training utilizes the Backpropagation Through Time (BPTT) algorithm, which applies backpropagation [37] to a time sequence. However, when dealing with long sequence inputs, RNN training may suffer from the common problems of vanishing and exploding gradients during backpropagation. This occurs because RNN builds very deep computational graphs by repeatedly applying the same operation at each time step of a long sequence. The LSTM and GRU are specialized recurrent neural network architectures designed to tackle these vanishing and exploding gradient issues. Memory cells in LSTM and GRU networks are equipped with gated units that dynamically control the flow of information, allowing the networks to “forget” old and unnecessary information and avoid the problems associated with multiplying large sequences of numbers during temporal backpropagation. GRU have the advantage of avoiding overfitting and reducing training time compared to LSTM, as GRU with two gates have significantly few parameters than LSTM with four gates.

**Figure 4:**
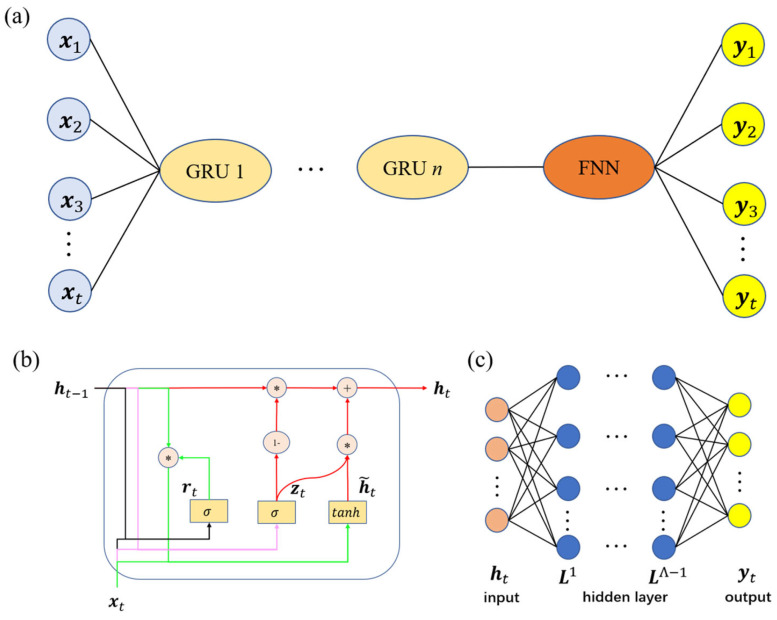
(**a**) GRU–FNN architecture; (**b**) details of GRU; (**c**) details of FNN.

To predict the time-dependent stress of viscoelastic materials, a surrogate model based on GRU and FNN (GRU–FNN) is employed, as illustrated in Figure 4a. At each time step, the GRU cell manages the information flow using a reset gate $r_{t}$, which regulates the integration of new input with the previous memory; and an update gate $Z_{t}$, which determines how much of the previous memory should be retained; and a hidden state $h_{t}$, which transfers information from the candidate hidden state ${\tilde{h}}_{t}$ forward, as depicted in Figure 4b. These two gates control the information flow between the long-term hidden state and the predictions at each time step. FNN then processes the final hidden state of the GRU as unidirectional flow signals, moving from the input layer through hidden layers to the output layer, as shown in Figure 4c. A detailed introduction to GRU and FNN will follow.

### 3.1. Gated Recurrent Unit

The reset gate output is [8,38]

(39) $$r_{t}=\sigma \left(W^{r}x_{t}+U^{r}h_{t-1}+b^{r}\right)$$

Here, the weight matrix $W^{r}$ connects the input at a specific time t to the current hidden layer within the reset gate, while the weight matrix $U^{r}$ represents the recurrent connection between the previous and current hidden layers within the reset gate. The initial hidden state is initialized the null vector $h_{0}=0$. $b^{r}$ denotes the bias vector, and σ refers to the activation function.

The update gate output is

(40) $$Z_{t}=\sigma \left(W^{z}x_{t}+U^{z}h_{t-1}+b^{z}\right)$$

where $W^{z}$, $U^{z}$, $b^{z}$ are respectively two weights matrices and a bias vector regulating the update mechanism in this gate.

The candidate hidden state ${\tilde{h}}_{t}$ is

(41) $${\tilde{h}}_{t}=\tanh\left[W^{h}x_{t}+U^{h}\left(r_{t}*h_{t-1}\right)+b^{h}\right]$$

where $W^{h}$ and $U^{h}$ are two different weight matrices, $b^{h}$ is a bias vector, the symbol ∗ denote the Hadamard product of two vectors with identical dimensions, tanh is the hyperbolic tangent function.

The GRU output current hidden state $h_{t}$ is

(42) $$h_{t}=\left(1-Z_{t}\right)*h_{t-1}+Z_{t}*{\tilde{h}}_{t}$$

where 1 is a unit vector, i.e., a vector filled with ones.

The total number of parameters $N_{P}$ in a GRU cell is related to the dimension $N_{I}$ of the input vector $x_{t}$ and the number of neurons $N_{H}$ in the hidden layers of the GRU cell, given by

(43) $$N_{P}=3N_{H}\left(N_{I}+N_{H}+2\right)$$

Note that the above bias vector is considered to consist of two different bias vectors related to two different weight matrices, respectively.

### 3.2. Feedforward Neural Network

The FNN can be described by the following compositional function [39]:

(44) $$O\left(h_{t},W,b\right)=\sigma^{\Lambda }\circ \sigma^{\Lambda -1}\circ \cdots \circ \sigma^{1}$$

where the symbol ∘ denotes the composition operator, the superscript Λ represents the total number of the neural network, and σ is the activation function with the following form:

(45) $$\sigma^{\lambda }\left(L^{\lambda -1}\right)=\sigma^{\lambda }\left(W^{\lambda }L^{\lambda -1}+b^{\lambda }\right),\;\lambda =1,\;\cdots ,\;\Lambda$$

Here, the superscript λ denotes the layer number of the neural network, $L^{\lambda -1}$ the input vector from the $\left(\lambda -1\right)\mathrm{th}$ layer (specially, $L^{0}=h_{t}$ and $L^{\Lambda }=y_{t}$), $W^{\lambda }$ and $b^{\lambda }$ respectively the weight matrices and biases vector of λth layer. Note that all hidden states of the last GRU are input into the same FNN in turn for training. The total parameters $N^{F}$ in FNN can be calculated as

(46) $$N^{F}=\sum_{\lambda =1}^{\Lambda }N^{\lambda }\left(N^{\lambda -1}+1\right)$$

where $N^{\lambda }$ is the number of neurons of the λth layer.

### 3.3. Training Procedure

The loss function, defined as the mean absolute error (MAE), is expressed as

(47) $$l\left(\theta \right)=\frac{1}{N^{D}}\sum_{k=1}^{m}\left|y_{k}-{\hat{y}}_{k}\right|$$

where $y_{k}$ and ${\hat{y}}_{k}$ denote the ground-truth and predicted outputs at the ith time sequence, $N^{D}$ is the total number of datapoints in the training dataset, and θ represents the parameters of the neural networks. These parameters are updated by minimizing the loss function, as follows:

(48) $$\theta^{*}=\underset{\theta }{argmin}\left(l\left(x,\theta \right)\right)$$

where $\theta^{*}$ represents the values of θ that minimize $l\left(x,\theta \right)$. Common optimization methods for minimizing the loss function include gradient decent techniques (such as Adam) and quai-Newton methods (such as L-BFGS). In this work, the Adam optimizer [40] is tentatively employed to update the hyperparameters. The Backpropagation (BP) technique and Backpropagation Through Time (BPTT) technique are used to compute the gradients of the loss function with respect to the parameters of FNN and GRU, respectively. In the following sections, the GRU–FNN model consists of three stacked GRU layers with 100 units each and a single time-distributed dense layer with 100 neurons. This configuration balances computational cost and error minimization through trial and error. The leaky rectified linear unit (LeakyReLU) is used as the activation function for the GRU, while the linear function is used for the FNN.

## 4. Results and Discussions

### 4.1. Initialization of GRU-FNN Parameters by Training Theoretical Data

The inputs and outputs are unrolled through 100 time steps, i.e., $x=\left\{x_{1},x_{2},\cdot \cdot \cdot ,x_{m}\right\}$ and $y=\left\{y_{1},y_{2},\cdot \cdot \cdot ,y_{m}\right\}$ with *m* = 100. Let $t_{k}$, $\lambda_{k}$ and $P_{k}$ denote the loading time, the stretch and the first P-K stress at the kth time step, respectively. $t_{k}$ is calculated by the following formula:

(49) $$t_{k}=\left\{\begin{array}{c} \frac{\lambda_{k}-1}{\left|\dot{\lambda }\right|},\;\dot{\lambda }>0\\ \frac{\lambda_{max}-1}{\left|\dot{\lambda }\right|}+\frac{\lambda_{max}-\lambda_{k}}{\left|\dot{\lambda }\right|},\;\dot{\lambda }<0 \end{array}\right.$$

where $\lambda_{max}$ is the maximum of λ during loading tests. The inputs $x_{k}=\left(t_{k},\lambda_{k}\right)$ and the outputs $y_{k}=\left(P_{k}\right)$ are used for the uniaxial loading-unloading viscoelasticity. Ten samples are generated by capturing 100 points per curve in Figure 2 and then split into training data (80% of total samples) and testing data (20% of total samples). The training data with a batch size of 2 are used during model’s training process, while the testing data are used for validation of the model’s generalization capabilities.

The GRU–FNN model is implemented in the software library Keras 2.10.0 and its information is listed in Table 1. In the output shape of layers, the first *None* refers to the batch, the second number the time steps and the third number the units (i.e., the dimension of the hidden state) of GRU or the dimension of the outputs of the dense layer. According to Equation (43), the numbers of parameters of GRU 1 and GRU 2 (GRU 3) layers are calculated respectively as 3 × 100 × (2 + 2 + 100) = 31,200 and 3 × 100 × (2 + 100 + 100) = 60,600. According to Equation (46), the number of parameters of the dense layer is calculated as 1 × (100 + 1) = 101.

**Figure 5:**
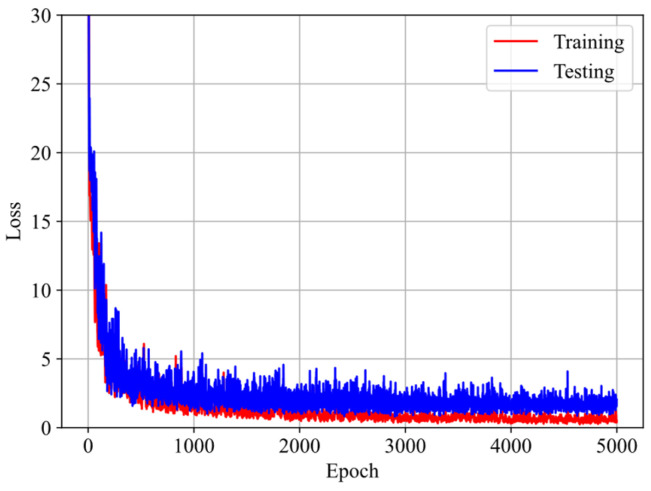
Evolution of the loss during training and testing with respect to epochs.

Figure 5 illustrates the evolution of loss with respect to epoch during training and testing. It is observed that the loss decreases dramatically at first and then stabilizes at a steady state. Additionally, the testing loss is slightly higher than the training loss. These observations suggest that the model does not suffer from overfitting or underfitting issues.

**Figure 6:**
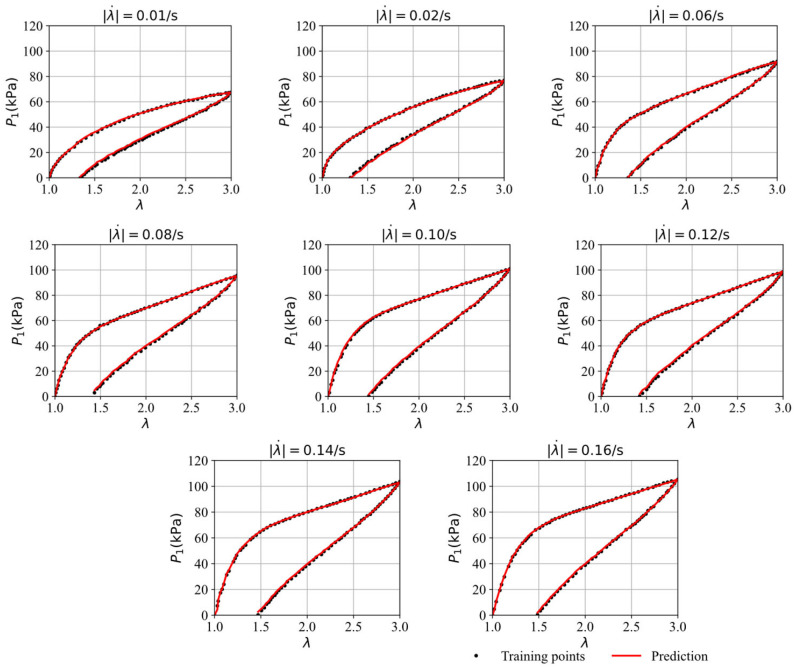
The comparison of the stress–strain curves from the training data and the model’s prediction after 5000 epochs.

Figure 6 compares the stress–stretch curves from the training data with the model’s predictions at different stretching rates after 5000 epochs. It was observed that the loss value of the training data stabilizes around 0.50 after approximately 5000 epochs through trial and error. Therefore, we trained the model for 5000 epochs. To evaluate the model’s accuracy under various conditions, the root mean square error (RSME) is employed, defined as $\mathrm{RMSE}=\frac{1}{m}\sum_{k=1}^{m}\sqrt{{\left(y_{k}-{\hat{y}}_{k}\right)}^{2}}$, where $y_{k}$ and ${\hat{y}}_{k}$ represents the ground truth and the predicted values. The RMSE quantifies the discrepancy between the predicted results and the training or testing data. RMSE values for all cases in this work are listed in Table 2. For the stretching rates of $\left|\dot{\lambda }\right|$ = 0.01/s, 0.02/s, 0.06/s, 0.08/s, 0.12/s, 0.14/s, and 0.16/s, the corresponding RMSE values are 0.76 0.56, 0.53, 0.71, 0.85, 0.83, 0.73, and 0.63, respectively. The predictions from the GRU–FNN model are highly consistent with training data, and the hysteresis curves exhibit typical viscoelasticity.

**Figure 7:**
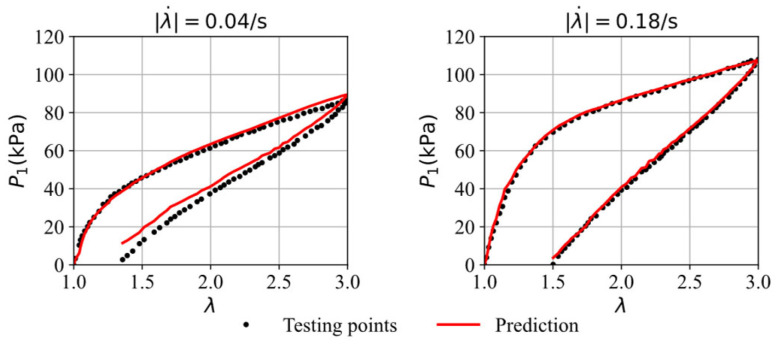
The comparison of the stress–strain curves from testing data and model’s prediction after 5000 epochs.

Figure 7 compares the stress–stretch curves from the testing data with the model’s predictions at two different stretching rates after 5000 epochs. For the stretching rates of $\left|\dot{\lambda }\right|$ = 0.04/s and 0.18/s, the corresponding RMSE values are 3.41 and 1.52, respectively. The testing data, which was not used during the training process, is accurately predicted by the trained model, demonstrating the GRU–FNN model’s strong generalization capabilities in predicting viscoelastic behaviors. The parameter values (weights and biases) here after 5000 epochs are saved for use as initial parameters in training the GRU–FNN model on experimental data.

### 4.2. Initialization of GRU–FNN Parameters by Training Theoretical Data of Viscoelasticity of VHB4905 with Scarce Data from Experiments

In the thermo-viscoelastic experiments of Liao and Hossain [41], VHB4905 samples with dimensions of 100 m × 10 mm × 0.5 mm are used for cyclic loading–unloading tests to investigate the time- and stretching rate-dependent viscoelasticity of VHB polymers. Three datasets, selected from the experimental stress–stretch curves at 273 K and three different stretching rates, i.e., 0.03/s, 0.05/s, and 0.10/s, are split into two training datasets for learning the viscoelastic behaviors and one testing dataset for checking the GRU–FNN model’s capabilities in predicting viscoelasticity of materials with scarce training data.

**Figure 8:**
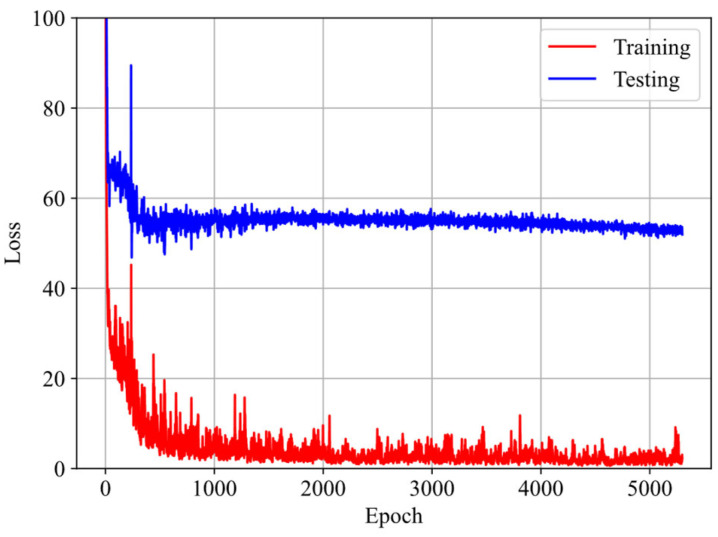
Evolution of loss with respect to epochs during training and testing at 273 K.

**Figure 9:**
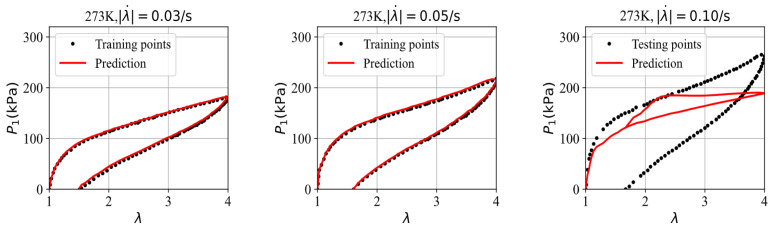
The comparison of the stress–stretch curves from the experimental data and the model’s prediction at 273 K after 5300 epochs.

First, the three datasets are used with the GRU–FNN model initialized with default parameter values (weights and biases), and the predicted stress is obtained after 5300 epochs. Figure 8 shows the evolution of the loss with respect to epochs during training and testing. It is observed that the loss for testing data are significantly higher than that for training data after several epochs and fluctuates at a high level, indicating model overfitting. Figure 9 compares the stress–stretch curves from the experimental data with the model’s predictions at 273 K after 5300 epochs. For the stretching rates of $\left|\dot{\lambda }\right|$ = 0.03/s, 0.05/s, and 0.10/s, the corresponding RMSE values are 2.84, 3.07, and 62.49, respectively. The training data from cyclic experiments at $\left|\dot{\lambda }\right|$ = 0.03/s and $\left|\dot{\lambda }\right|$ = 0.05/s are predicted accurately. However, the testing data from cyclic experiments at $\left|\dot{\lambda }\right|$ = 0.10/s show substantial divergence with the predicted results, further demonstrating the poor generalization capabilities of the model in predicting viscoelasticity of materials with scarce training data.

**Figure 10:**
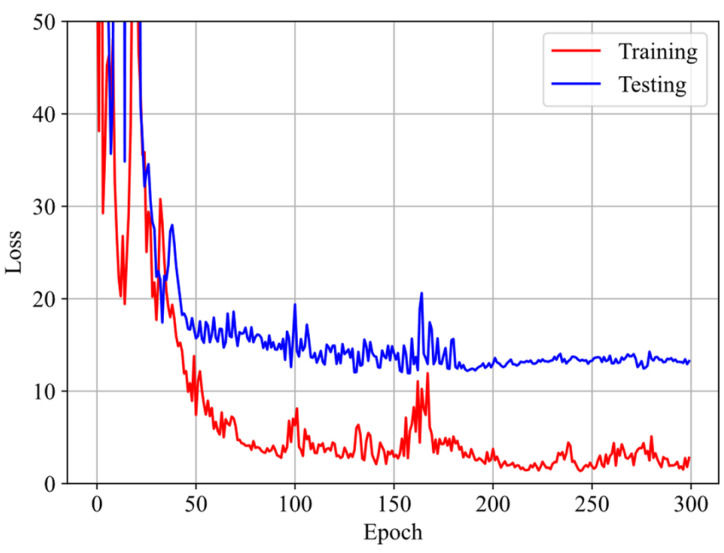
Evolution of the loss with respect to epochs during training and testing at 273 K.

**Figure 11:**
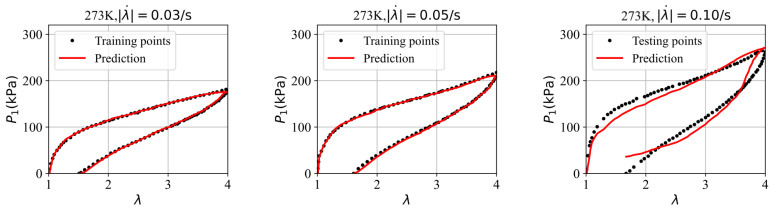
The comparison of the stress–strain curves from the experimental data and the model’s prediction at 273 K.

To address the overfitting issues associated with scarce data, the parameter values from training the GRU–FNN model with theoretical data are used as the initialization for training on the experimental data. Figure 10 shows the evolution of the loss with respect to epochs during both training and testing. It is evident that the gap between the loss for training and testing data has significantly narrowed compared to Figure 8. Given that the theoretical data were trained for 5000 epochs, the experimental data were trained 300 epochs to maintain consistency in the total training duration (5300 epochs). Figure 11 compares the stress–stretch curves from the experimental data with the model’s predictions at 273 K after 300 epochs. It was also observed that the loss values of training data and testing data stabilize after approximately 300 epochs through trial and error. For the stretching rates of $\left|\dot{\lambda }\right|$ = 0.03/s, 0.05/s, and 0.10/s, the corresponding RMSE values are 1.90, 2.62, and 15.70, respectively. The high degree of consistency between the predicted and experimental stress–stretch curves indicates that our approach has effectively alleviated the overfitting issues.

**Figure 12:**
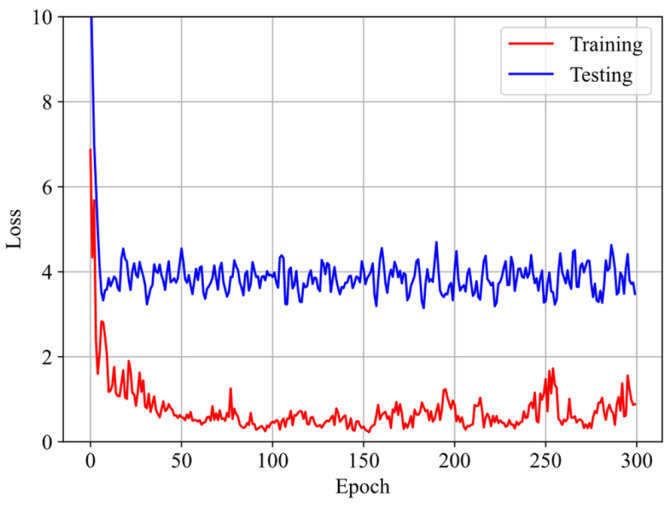
Evolution of loss with respect to epochs during training and testing at 313 K.

**Figure 13:**
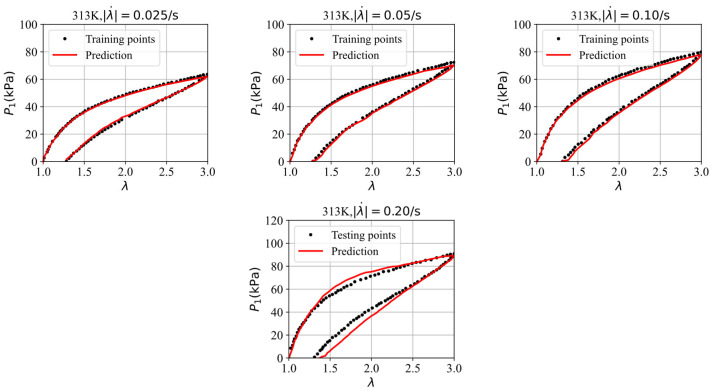
The comparison of the stress–strain curves from the experimental data and the model’s prediction at 313 K.

**Figure 14:**
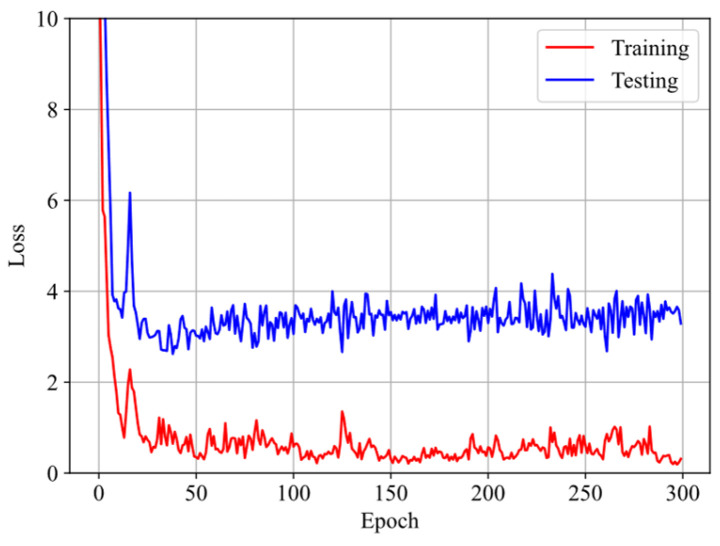
Evolution of loss with respect to epochs during training and testing at 333 K.

**Figure 15:**
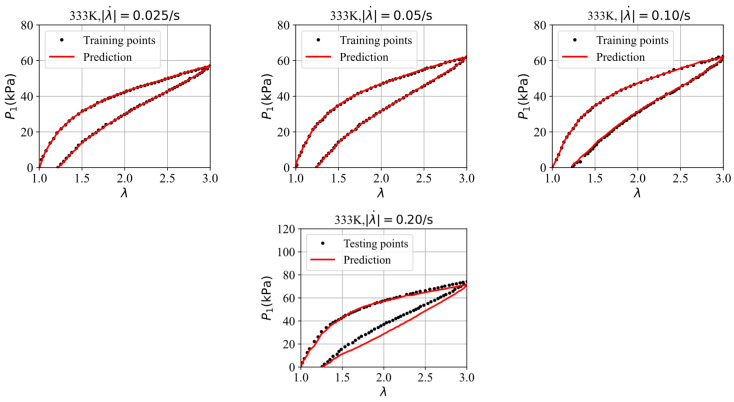
The comparison of the stress–strain curves from the experimental data and the model’s prediction at 333 K.

Finally, we split four datasets, selected from the stress–stretch curves at four different stretching rates, i.e., 0.025/s, 0.05/s, 0.10/s, and 0.20/s in the experiments of VHB4905 samples with dimensions of 130 mm × 10 mm × 0.5 mm [42], into three training datasets and one testing dataset to further assess the GRU–FNN model’s capabilities in predicting the viscoelasticity of materials across different temperatures. The saved parameter values from training with theoretical data are used for initialization. Figure 12 shows the evolution of the loss with respect to epochs during training and testing at 313 K. It can be seen that the gap of the loss during training and testing is smaller than that observed in Figure 9 due to the inclusion of an additional training dataset. Figure 13 compares the stress–stretch curves from the experimental data and the model’s predictions at 313 K after 300 epochs. For the stretching rates of $\left|\dot{\lambda }\right|$ = 0.025/s, 0.05/s, 0.10/s, and 0.20/s at 313 K, the corresponding RMSE values are 0.81, 1.32, 1.72, and 4.55, respectively. Figure 14 shows the evolution of the loss with respect to epochs during training and testing at 333 K. The evolution trend is similar with that in Figure 12. Figure 15 compares the stress–stretch curves from the experimental data and the model’s predictions at 333 K after 300 epochs. At 333 K, for the same stretching rates, the RMSE values are 0.24, 0.31, 0.53, and 4.27, respectively. The model accurately predicts the training data and provides reasonable predictions for the testing data, demonstrating improved generalization capabilities.

**Table 2:** Statics of RMSE values.

Cases	Stretching Rates (/s)	RMSE Values (kPa)
Figure 6	(0.01,0.02,0.06,0.08,0.10,0.12,0.14,0.16)	(0.76,0.56,0.53,0.71,0.85,0.83,0.73,0.63)
Figure 7	(0.04,0.18)	(3.41,1.52)
Figure 9	(0.03,0.05,0.10)	(2.84,3.07,62.49)
Figure 11	(0.03,0.05,0.10)	(1.90,2.62,15.70)
Figure 13	(0.025,0.05,0.10,0.20)	(0.81,1.32,1.72,4.55)
Figure 15	(0.025,0.05,0.10,0.20)	(0.24,0.31,0.53,4.27)

### 4.3. Analyzing the Sensitivity of the GRU-FNN Model

**Figure 16:**
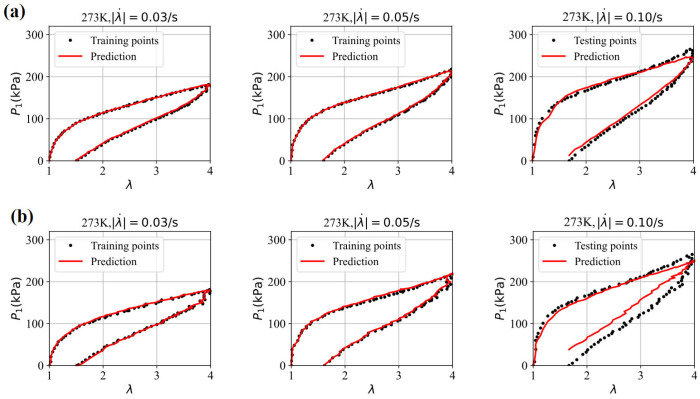
The comparison of the stress–strain curves from the model’s prediction at 273 K and the experimental data with (**a**) 0.5% noise and (**b**) 1.0% noise.

To test the sensitivity of the GRU–FNN model to input data, a random number is generated from a standard normal distribution (mean = 0, standard deviation = 1). The generated noise value is then multiplied by 0.5% or 1.0% of the original stretch values, resulting in a noise adjustment value that is proportional to the magnitude of the original data. This value is added to the original data to simulate noise in the data. Since the time series can be calculated based on the stretching strain and stretching rate, we only apply noise processing to the stretching strain sequence here. The predicted results of the dataset at 273 K with added noise are shown in Figure 16. It can be observed that the model is resilient to small amounts of noise. However, as the noise increases, the prediction error also increases. When the noise exceeds the error between adjacent points, the prediction curve tends to become unsmooth and may even change direction, leading to a complete failure of the prediction. The same pattern was also observed in the predictions of the other two datasets at 313 K and 333 K, respectively, which are not shown here, but the corresponding RMSE values are listed in Table 3.

## 5. Conclusions

In this paper, a physics-guided GRU–FNN model for predicting the viscoelasticity of solids at large deformation is proposed and validated by the comparison of the stress–stretch curves from the model prediction and the uniaxial experiments of VHB4905. The major novelty of the present work lies in the following aspects. First, the time- and loading path- dependent stress can be predicted by utilizing both time and stretch/strain sequences as inputs of the proposed GRU–FNN model. Second, the saved values of the parameters after training theoretical data are employed as the parameter initialization of GRU–FNN model for learning the viscoelastic behavior of solids with scarce experimental data, which significantly alleviates the overfitting issues that arise in the case of scarce data. The results show that the GRU–FNN model can reduce the RMSE by several times, significantly improving its generalization ability in scarce data scenarios. The physics-guided initialization of parameters in this work provides a new idea on how to incorporate physical knowledge into a machine learning model when the governing equations describing physical phenomenon in one material cannot be specified. The model can be applied to predict multiaxial stress–strain relations of viscoelastic solids in a similar manner when the experimental data from multiaxial tests are available, which will be our future study subject. This study facilitates the rapid prediction of materials’ nonlinear viscoelastic properties and lays a theoretical foundation for future real-time monitoring and performance prediction of polymer-based devices such as electronic artificial muscles and dielectric elastomer actuators.

## Figures and Tables

**Table 1:** Information of the GRU-FNN model.

Layer	Output Shape	Parameters
GRU 1	(*None*, 100,100)	31,200
GRU 2	(*None*, 100,100)	60,600
GRU 3	(*None*, 100,100)	60,600
Dense	(*None*, 100,1)	101

**Table 3:** Statics of RMSE values.

Cases with Noise	Stretching Rates (/s)	RMSE Values (kPa)
273 K_0.5%	(0.03,0.05,0.10)	(1.93,2.17,8.28)
273 K_1.0%	(0.03,0.05,0.10)	(2.16,3.80,20.30)
313 K_0.5%	(0.025,0.05,0.10,0.20)	(0.45,0.59,0.84,4.55)
313 K_1.0%	(0.025,0.05,0.10,0.20)	(1.84,1.23,0.83,4.73)
333 K_0.5%	(0.025,0.05,0.10,0.20)	(1.37,0.84,0.66,5.27)
333 K_1.0%	(0.025,0.05,0.10,0.20)	(0.50,0.54,0.59,5.16)

## Data Availability

The original contributions presented in the study are included in the article, further inquiries can be directed to the corresponding author.
